# An XGBoost-based predictive framework for diabetes mellitus multi-classification

**DOI:** 10.1038/s41598-026-65844-3

**Published:** 2026-08-14

**Authors:** Moataz M. El Sherbiny, Mohamed G. Abdelfattah, Ali E. Takieldeen, Hossam El-Din Mostafa, Hala B. Nafea

**Affiliations:** 1 Department of Electronics and Communication Engineering, Faculty of Engineering, Mansoura University, Mansoura, Egypt; 2 Faculty of Artificial Intelligence, Horus University, New Damietta, Egypt; 3 Department of Cyber Security, Faculty of Artificial Intelligence, Delta University, Mansoura, Egypt

**Keywords:** Diabetes mellitus, Multi-classification, Preprocessing, Class imbalance, Extreme gradient boosting, Performance evaluation, Computational biology and bioinformatics, Diseases, Health care, Mathematics and computing

## Abstract

Diabetes mellitus is a persistent metabolic condition that requires accurate and early diagnosis to prevent severe complications. This paper proposes an Extreme Gradient Boosting (XGBoost)-based predictive framework for multi-classification of diabetes mellitus into non-diabetic, pre-diabetic, and diabetic classes. After standardization and exclusion of non-clinical identifiers, Duplicate clinical records were removed from the original dataset, leaving 826 unique records. Comprehensive preprocessing pipeline used a stratified 70:30 train-test split and five-fold cross-validation; scaling and resampling were performed only within training partitions. Experimental results on the original dataset XGBoost achieved an accuracy of 99.60%. Both Random Over Sampling (ROS) and Syntenic minority over sampling technique (SMOTE) have also achieved 99.60% accuracy but provided improved generalization at the expense of higher computational cost. In contrast, Random Under Sampling (RUS) and Cluster Centroids (CC) reduced accuracy to 92.74% and 90.32%, respectively due to information loss. Across five folds, the original XGBoost model achieved 98.79 ± 1.13% accuracy. Benchmarking against Logistic Regression, Random Forest, Support Vector Machine, Decision Tree, and K-Nearest Neighbors showed that XGBoost provided the strongest performance. These findings highlight the effectiveness of XGBoost while emphasizing classification accuracy in multiclass diabetes prediction systems.

## Introduction

Diabetes poses major risks to various body organs since it reduces the ability to extract energy from consumed food. It is considered as a disorder that elevates the insulin level in blood beyond the normal threshold^1^. Diabetes is regarded as an invisible illness, with around 240 million undiagnosed cases worldwide, or 1 in 2 individuals diagnosed with the condition^2^. The condition is linked to harsh consequences such as cardiovascular problems, renal failure, neuropathy, and retinopathy^3^, Such risks affect quality of life and elevate mortality rates. Three types of diabetes are listed as follows: Type-1 Diabetes, Type-2 Diabetes and Gestational Diabetes. They are different in threats, causes, symptoms as well as treatment. Type 1 diabetes is found in nearly 10–15% of all diabetic conditions. This type of diabetes generally shows up in the childhood or adolescent stage^4^. Type 2 diabetes is more related to an older stage and is defined as irregular glucose secretion owing to peripheral resistance and is responsible for 85–90% of patients affected with hyperglycemia^5^. Clinical appearance and development differ significantly, and few patients might not be categorized as having either type 1 or 2 at first. Therefore, it makes the diagnosis accurate only when time passes. Gestational diabetes is a form of diabetes that develops typically around the 24th week of pregnancy. Pregnant individuals may experience common symptoms such as increased thirst, fatigue, and frequent urination. This is due to non-sufficient insulin to regulate blood glucose levels efficiently. Most cases resolve after childbirth but increase the risk of developing type 2 diabetes later in life^6^.

Timely diagnosis and efficient care are essential in reducing the progression of diabetes and its associated consequences. Machine learning (ML) serves as a revolutionary tool in disease diagnosis and classification. ML facilitates data-driven analysis of clinical patterns that may not be easily discernible by conventional statistical methods. Notably supervised learning methodologies have exhibited robust efficacy in both binary and multiclass classification problems. Consequently, machine learning is crucial in enhancing diagnostic efficiency, minimizing human error, and facilitating scalable, intelligent healthcare systems. Limitation extended to many missing values in available datasets which can be misleading to the machine learning classification algorithms. Therefore, proper preprocessing is a very vital and indispensable process. Finding organized data in electronic format is extremely difficult, and the process of transferring manually recorded data to electronic form takes a long period of time. Moreover, many hospitals and clinics are not willing to share data for purposes of patient privacy and confidentiality.

## Literature review

Most recent research focuses on a binary classification method for prediction of diabetes due to limited availability of datasets. These related studies have been conducted on the same dataset named Pima Indian Diabetes Dataset (PIDD). It is recognized as one of the most prominent datasets for binary classification of diabetes via machine learning techniques. It contains many missing values and outliers which could be misleading to the training process of machine learning models. The significance of artificial intelligence and machine learning in reshaping medical practices is evidenced by the works of researchers. The summary of their reported findings is presented in Table 1.

**Table 1:** Summary of related work.

Author	Methodology	Model	Accuracy (%)
Barik et al.^7^	Binary classification	XGB	74.1
Mushtaq et al.^8^		RF	80.7
Kangra et al.^9^		NB	74
Mosa et al.^10^		NB and KNN	79 and 69
R. Ashisha et al.^11^		RF	92
Maydanchi1 et al.^12^		XGB	97
Sherbiny et al.^13^		RF, XGB and LR	96.88
Rajput et al.^14^	Multi-classification	DT and GB	89
Altalabani et al.^15^		SVM and KNN	95.4 and 89.9
Ghosh et al.^16^		RF	98.22
Tiwari et al.^17^		XAI	99

Barik et al.^7^ introduced two categories of machine learning methodologies for diabetes prediction. Their research utilized the Pima Indian Diabetes dataset. The predictive accuracy for Random Forest was 71.9%, however XGBoost achieved a superior accuracy of 74.1%. Mushtaq et al.^8^ emphasized the need of class balancing for diabetes prediction. They utilized a two-phase model selection process. Logistic Regression, Support Vector Machine, K-Nearest Neighbors, Gradient Boosting, Naive Bayes, and Random Forest were utilized to assess the efficacy of predictive models. Random Forest demonstrated the highest accuracy of 80.7% following the application of SMOTE. The combination of the top three models achieved 82% and 81.7% in terms of accuracy on unbalanced dataset and balanced dataset respectively. Kangra et al.^9^ employed several supervised machine learning methodologies for diabetes prediction: Naive Bayes, K-Nearest Neighbors, Support Vector Machines, and Logistic Regression. The experiment was conducted on the PIDD. The classification accuracies are as follows: NB achieved 72.6%, KNN attained 66.1%, DT recorded 71.8%, and RF reached 64.9%. The dataset was partitioned into training and testing subsets via tenfold cross-validation throughout the preprocessing phase. This research contrasted the employed machine learning algorithms using three assessment metrics: accuracy, precision, and recall. Results demonstrated that SVM attained the greatest result of 74.3% accuracy, followed closely by LR with a score of 74%. Mosa et al.^10^ presented a predictive framework based on a supervised machine learning algorithm. The authors implemented both Naïve Bayes (NB) and K-nearest Neighbor (KNN) in their suggested model. They achieved accuracies of 79% and 69% respectively on the Pima Indian Diabetes dataset. Ashisha et al.^11^ established a model that equilibrates class distribution. They employed random oversampling technique in their research. A deep analysis of various machine learning algorithms is conducted. Random Forest outperformed the other classifiers by reaching 92% accuracy. Maydanchi et al.^12^ implemented nine machine learning methodologies. The results were evaluated based on accuracy, precision, F1-score, recall, and runtime. Gradient boosting had a maximum accuracy of 97% in 144.42 s. Extreme gradient boosting achieved same accuracy in a much-reduced time of about 4.1 s. Sherbiny et al.^13^ proposed an ensemble model for diabetes diagnosis combining Random Forest, XGB, and Logistic Regression. The approach leveraged the strengths of multiple algorithms to improve classification performance. The model achieved a high accuracy of 96.88% and precision of 89.85%. Rajput et al.^14^ adopted a series of supervised machine learning methodologies, including logistic regression, decision trees, random forests, Naïve Bayes, and gradient boosting. The decision tree achieved a maximum F1-score of 89.67%. Gradient boosting attained a recall of 81% and a precision of 98.85%. The results demonstrated that the decision tree and gradient boosting strategies surpassed the others, achieving a balanced accuracy of 89%. Altalabani et al.^15^ focused on the multiclass classification of diabetes. They employed various machine learning methodologies. SVM achieved a training accuracy of 95.4%, surpassing the K-nearest neighbor, which recorded 89.9% for both training and testing. Ghosh et al.^16^ implemented preprocessing techniques including outlier elimination, feature normalization, and hyperparameter optimization. They utilized logistic regression, support vector machines, decision trees, and random forests on a multiclass dataset. The findings indicated that Random Forest attained the maximum accuracy of 98.22%. Tiwari et al.^17^ developed explainable multiclass diabetes-risk stratification models and incorporated five-fold cross-validation and SHAP analysis. A recent healthcare review by Sadeghi et al.^18^ emphasized that transparent and interpretable models are particularly important in safety–critical clinical settings.

**Fig. 1:**
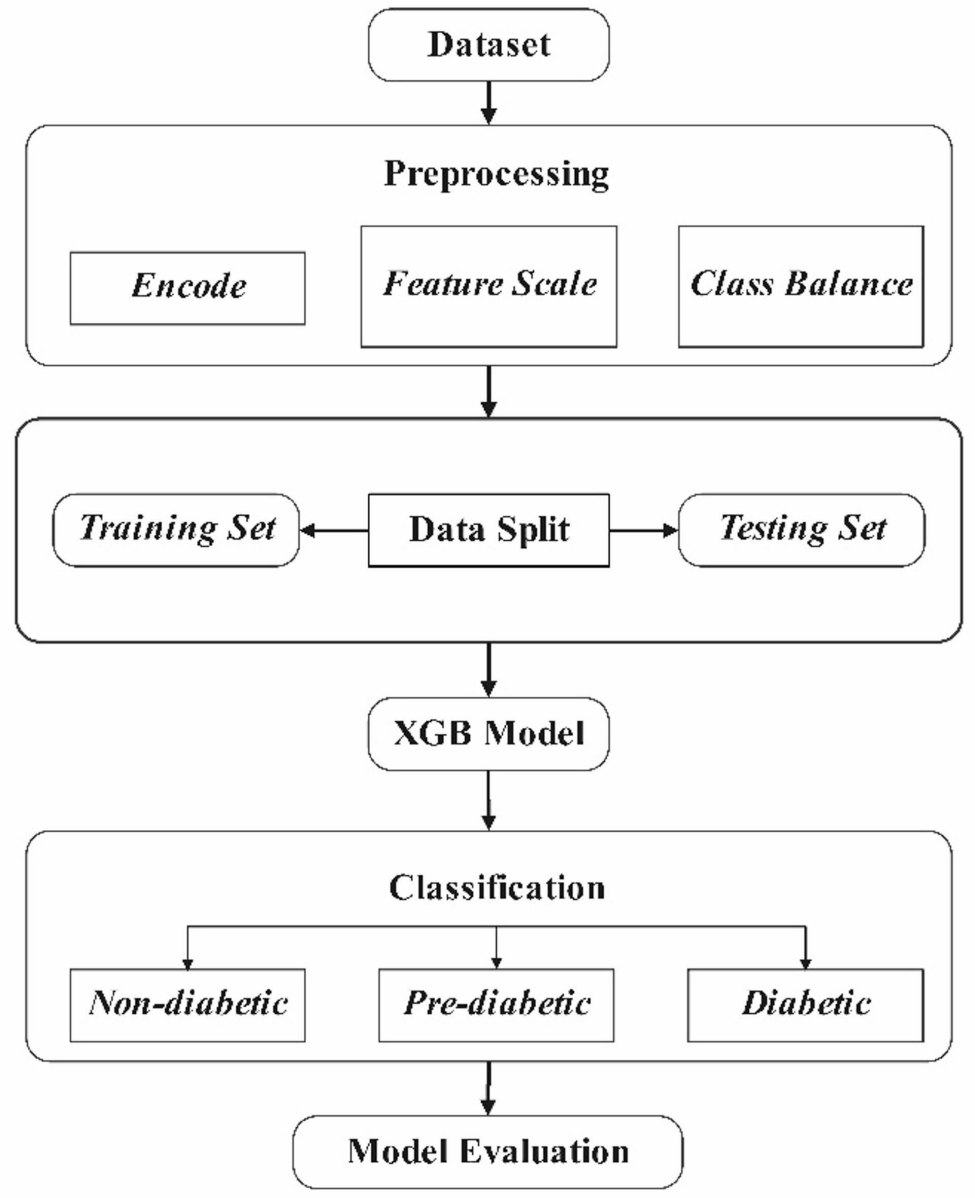
Workflow of the proposed multiclass diabetes-classification framework.

Despite these advances, comparatively few studies evaluate diabetic multiclassification while simultaneously comparing multiple balancing methods, benchmarking conventional classifiers, reporting computational runtime, and assessing stability through cross-validation. Therefore, this study addresses that gap by evaluating six classifiers under original, ROS, SMOTE, RUS, and Cluster Centroids training conditions using both a stratified train-test split and stratified five-fold cross-validation (Fig. 1).

### Contributions

The paper’s key contributions are stated below:

- The primary aim of this study is to employ comprehensive preprocessing to handle unscaled heterogenous data and class imbalance.
- A transparent preprocessing procedure standardizes categorical labels, excludes non-related clinical fields, removes duplicate clinical records before model evaluation.
- An experimental design fits scaling and class-balancing operations only on training data and retains the holdout test set unchanged.
- XGBoost is evaluated under original, ROS, SMOTE, RUS, and Cluster Centroids conditions and is benchmarked against Logistic Regression, Random Forest, Support Vector Machine, Decision Tree, and K-Nearest Neighbors.
- Performance is reported using a stratified 70:30 for training and testing respectively split in addition to five-fold cross-validation. The original XGBoost model achieved 99.60% holdout accuracy and 98.79 ± 1.13% cross-validated accuracy.

The subsequent sections of the paper are systematically arranged in a sequential manner: In the “Literature review” section, examine related prior studies. The “Methodology” section outlines the proposed approach, while the “Discussion” section will give the results and commentary. “Conclusion” section delineates the conclusion of this paper.

## Methodology

The type of dataset profoundly influences the selection of machine learning methods, ensuring that the analysis corresponds to the specific challenges of the medical sector. Datasets used in healthcare fields can be classified into clinical data, medical images and time series. Each type necessitates a specialized machine learning approach for optimal analysis. Clinical data are often organized in tabular structure typically in .xslx or .csv formats. Such data encompasses patient demographics and laboratory results, rendering it more suitable for supervised techniques in classification. Image datasets such as X-rays and MRIs require convolutional neural networks (CNNs) for feature extraction and classification, underscoring the significance of artificial intelligence in medical imaging diagnoses. Moreover, time-series data gathered from electronic health records recurrent neural networks (RNNs) to model temporal trends.

Labeled data is a crucial part for supervised machine learning classification tasks, as they provide the essential input–output pairs that algorithms need to learn patterns and relationships^19^. Medical datasets frequently exhibit problems such as heterogeneous data and class imbalance. These issues have a severe impact on the efficacy of learning systems. Data preprocessing is an essential aspect in deployment of prediction models, as it directly impacts learning efficiency and model generalization. To address these challenges, systematic preprocessing procedures are applied to transform the original dataset into a format suitable for machine learning models.

Categorical attributes must be systematically encoded into numeric formats to ensure compatibility with machine learning algorithms. Consequently, the encoding constitutes a critical preprocessing step, as it enables effective data interpretation and computational processing by learning models^20^. In this study, label encoder is deployed to represent non-numeric attributes by assigning a unique integer value to each category^21^. It is considered as a computational-efficient approach. Since, it does not increase the dimensionality of the dataset. Controversy, machine learning algorithms may misinterpret encoded values as having quantitative significance. Therefore, rescaling data is a must since machine learning models are biased towards features having large scale.

A dataset is balanced when its classes contain approximately equal numbers of observations. However, this condition is rarely satisfied in many practical tabular datasets such as medical records. The efficacy of machine learning models determined by accuracy becomes unreliable. Since they are biased towards the majority class. Furthermore, Minority class patterns may be under-learned. Class-balancing methods modify the training distribution to improve minority-class learning. Random oversampling (ROS) and the Synthetic Minority Oversampling Technique (SMOTE) increase minority-class representation, whereas random undersampling (RUS) and Cluster Centroids (CC) reduce the majority class. In this study, every sampler was applied exclusively to the training set and separately within each cross-validation training fold.

ROS duplicates minority class samples randomly till achieving balance. One of its key advantages is that it preserves the original data. On the other hand, the risk of overfitting increases by increasing the dataset size and training time since no new information is introduced. SMOTE generates synthetic minority samples by interpolating between existing minority samples and their nearest neighbors^22^. However, the generation of unrealistic samples could be considered as a misleading training parameter.

RUS removes random samples from the majority class to match the size of minority class^23^. It is quite simple straightforward technique known for its computational efficiency since it reduces the training time. It is quite effective for comparatively large datasets. Nevertheless, there exists a risk of forfeiting potential information. Model generalization may deteriorate where some informative samples may be eliminated. CC maintains all minority class samples unaltered while employing cluster centers to summarize the majority class rather than deleting majority class instances at random^24^.

Machine learning (ML) enables machines to learn patterns from data using mathematical and statistical models without explicit programming. ML algorithms are categorized into supervised and unsupervised learning. Supervised learning utilizes labeled datasets, where input–output pairs are provided to train models for either classification or regression tasks. Classification predicts categorical outcomes, such as identifying whether a patient is diseased or non-diseased, whereas regression estimates continuous variables, such as blood pressure levels or disease progression. In contrast, unsupervised learning operates without labeled outputs and primarily focuses on clustering techniques to uncover hidden structures or relationships within data. Extreme Gradient Boosting (XGBoost) is one of the most prominent machine learning models utilized in disease classification problems. Extreme XGBoost is an ensemble-learner algorithm derived from gradient boosting decision trees^25^. It is able to detect non-linear feature interactions, handle varied clinical factors, and integrate regularization. XGBoost effectively models such parameters throughout the sequential construction of decision trees. XGBoost minimizes classification error via gradient-based optimization through integrating relatively weaker and simpler models. For classification tasks, including binary and multiclass problems. For a sample i and class c, the multiclass softmax probability is defined in Eq. (1).

1 $$P\left( {y = cx} \right) = \frac{{\exp \left( {\hat{y}_{c} } \right)}}{{\mathop \sum \nolimits_{1}^{i} {\mathrm{exp}}(\hat{y}_{i} )}}$$

**Fig. 2:**
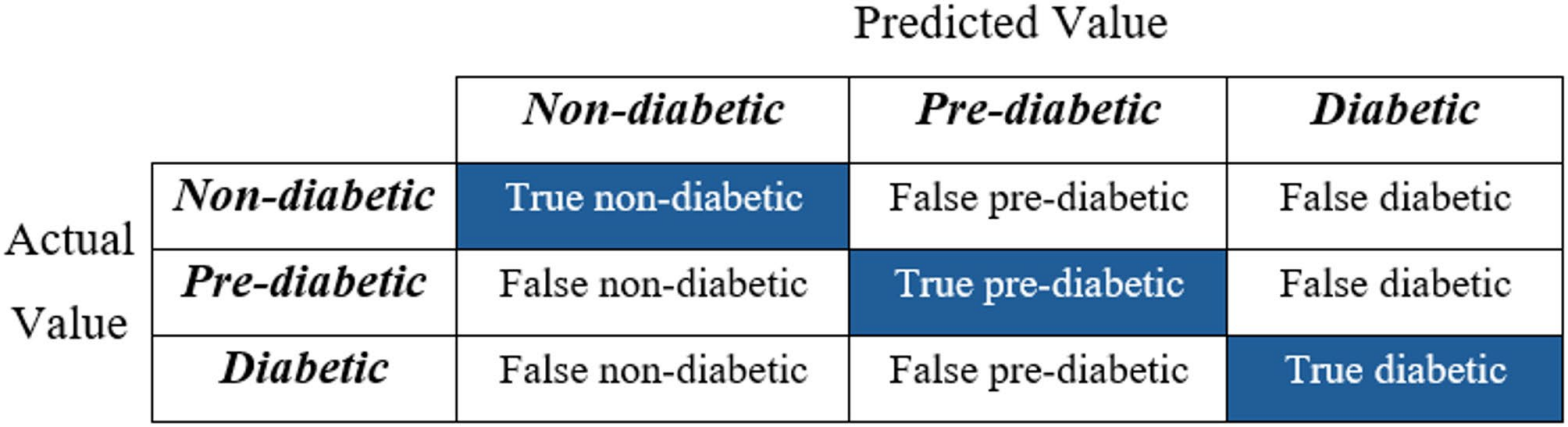
Structure and interpretation of the three-class confusion matrix.

The prominent features to evaluate the performance of deployed model are accuracy, precision, recall, F1- score and run time. The number of classes is indicated as “n”, while the number of true positives (TP), false positives (FP), true negatives (TN), and false negatives (FN) are the four factors needed to calculate these performance metrics as demonstrated in Eqs. (2–5)^26,27^. These values are summarized in confusion matrix as shown in Fig. 2. Runtime was also measured as the elapsed time required to fit the pipeline and generate predictions.

2 $$Accuracy=\frac{\sum_{i=1}^{n}{TP}_{i}}{\sum_{i=1}^{n}{(TP}_{i}+ {TN}_{i} + {FP}_{i}+{FN}_{i)}}$$

3 $${Precision}_{i}=\frac{{TP}_{i}}{{TP}_{i} + {FP}_{i}}$$

4 $${Recall}_{i}=\frac{{TP}_{i}}{{TP}_{i} + {FN}_{i}}$$

5 $${F1-score}_{i}=2*\frac{{Precision}_{i} *{ Recall}_{i}}{{Precision}_{i} + {Recall}_{i}}$$

### Model and results

The code-script is written in Python-Programming language on Kaggle Jupyter Notebook. Pandas and NumPy were used for data handling, Matplotlib for visualization, scikit-learn for preprocessing and benchmark classifiers, imbalanced-learn for resampling pipelines, and XGBoost for the proposed classifier. The notebook records the installed library versions at runtime to support reproducibility. The primary functions of the imported libraries are summarized in Table 2.

**Table 2:** Principal functions of the software libraries used in the implementation.

Library	Function
Pandas	Data manipulation, loading, and preprocessing data
NumPy	Handling numerical operations and arrays
Matplotlib	Plotting graphs and data visualization
Scikit-learn	Implementing machine learning algorithms, preprocessing, model evaluation, and metrics
Imbalanced-learn	Implementing ROS, SMOTE, RUS, Cluster Centroids, and leakage-controlled resampling pipelines

### Dataset description and data quality assessment

The data were gathered from the Medical City Hospital laboratory and the Specialized Center for Endocrinology and Diabetes et al.-Kindy Teaching Hospital in Iraq^28^. The source file contained 1000 records and 14 columns: 11 clinical predictors, the target class, and two identifier fields (ID and No_Pation). All available records were initially eligible for analysis. The source repository does not provide detailed patient-level inclusion and exclusion criteria; therefore, this limitation is stated explicitly. No missing values were detected. After excluding the two identifier fields from duplicate comparison, 174 repeated clinical records were identified and removed before any data split. The final dataset contained 826 unique records: 96 non-diabetic, 40 pre-diabetic, and 690 diabetic cases. The class-specific demographic summary is presented in Table 3. The dataset comprises medical information and laboratory analysis such as level of urea and cholesterol in blood, average level of glucose and body mass index as described in Table 4.

**Table 3:** Class distribution and demographic characteristics after duplicate-record removal.

Class	n	Percentage (%)	Age, mean ± SD	Age range	Female	Male
Non-diabetic	96	11.62	44.27 ± 9.45	25–77	58	38
Pre-diabetic	40	4.84	43.40 ± 7.66	30–55	13	27
Diabetic	690	83.54	55.36 ± 7.48	20–79	292	398
Overall	826	100.00	53.49 ± 8.81	20–79	363	463

**Table 4:** Clinical predictors and target variable used in the analysis.

Feature	Description
Gender	Categorical value that represents the sex of individual (Male “1”—Female “0”)
AGE	Age of person (years)
Urea	Level of urea in blood (mg/dL)
Cr	Level of creatine (mg/dL)
HbA1c	Level of average glucose level over the last three months expressed as percentage
Chol	Total cholesterol in the blood (mg/dL)
TG	Triglycerides measure the amount of fat in blood (mg/dL)
HDL	High Density Lipoprotein is a measure of good cholesterol
LDL	Low Density Lipoprotein is a measure of bad cholesterol
VLDL	Very Low-Density Lipoprotein is a measure of another bad cholesterol that carries triglycerides
BMI	Body mass index is a measure of body fat based on height and weight (kg/m^2^)
CLASS	Target label for diabetes (Non-diabetic “0”—Pre-diabetic “1”—Diabetic “2”)

Gender and class labels were standardized before modeling. Gender was encoded as female = 0 and male = 1, and the target classes were encoded as non-diabetic = 0, pre-diabetic = 1, and diabetic = 2. The ID and No_Pation columns were excluded from all model inputs.

**Fig. 3:**
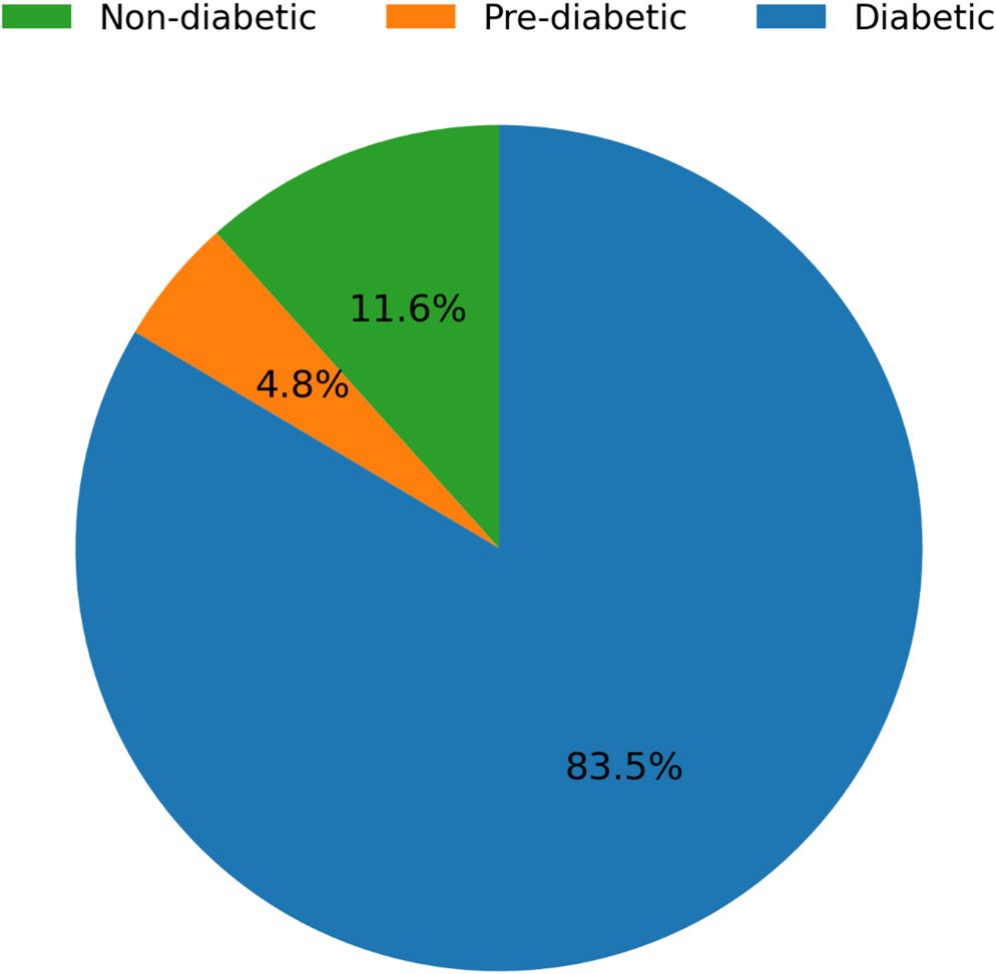
Class distribution of the 826 unique records.

First, the target distribution shown in Fig. 3 demonstrates a critical necessity for class balance. Where there are 96 non-diabetic cases, 40 cases are predicted to be diabetic, and 690 actually diabetic patients. Standardization was applied using StandardScaler. To prevent information leakage, the scaler was fitted only on the training data and then applied to the corresponding validation or test data. Within each imbalanced-learn pipeline, scaling preceded the selected sampler so that distance-based resampling operated on comparable feature scales.

For the evaluation, the 826 unique records were divided into 70% training and 30% testing partitions at random_state = 42. The test set remained completely unchanged. ROS, SMOTE, RUS, and Cluster Centroids were applied only to the training data. Model robustness was additionally assessed using stratified five-fold cross-validation with shuffling data. Despite being a quick straightforward technique, the findings might vary with different seeds.

Fifth, model was trained on extreme gradient boosting which is recognized for its efficacy in handling missing values within datasets. XGBoost are exceptionally proficient at predicting tasks. They continually attain enhanced accuracy by methodically rectifying the mistakes of preceding models. Furthermore, it may optimize various loss functions and include regularization methods to mitigate overfitting. Scalability is among the key features of XGBoost, where it directs abrupt learning through parallel and distributed computing as well as providing well-structured memory usage^29^. The selected hyperparameters and their meanings are summarized in Table 5.

**Table 5:** XGBoost hyperparameters used in all experiments.

Hyperparameter	Meaning	Selected value
n_estimators	Number of sequential boosting trees	100
learning_rate	Contribution of each tree to the ensemble	0.30
max_depth	Maximum depth of each tree	6
subsample	Fraction of training records used per tree	1.0
colsample_bytree	Fraction of features used per tree	1.0
objective	Three-class probabilistic objective	multi:softprob
eval_metric	Internal evaluation metric	mlogloss
random_state	Seed for reproducibility	42
tree_method	Tree-construction algorithm	hist
n_jobs	Number of processing threads	1

**Fig. 4:**
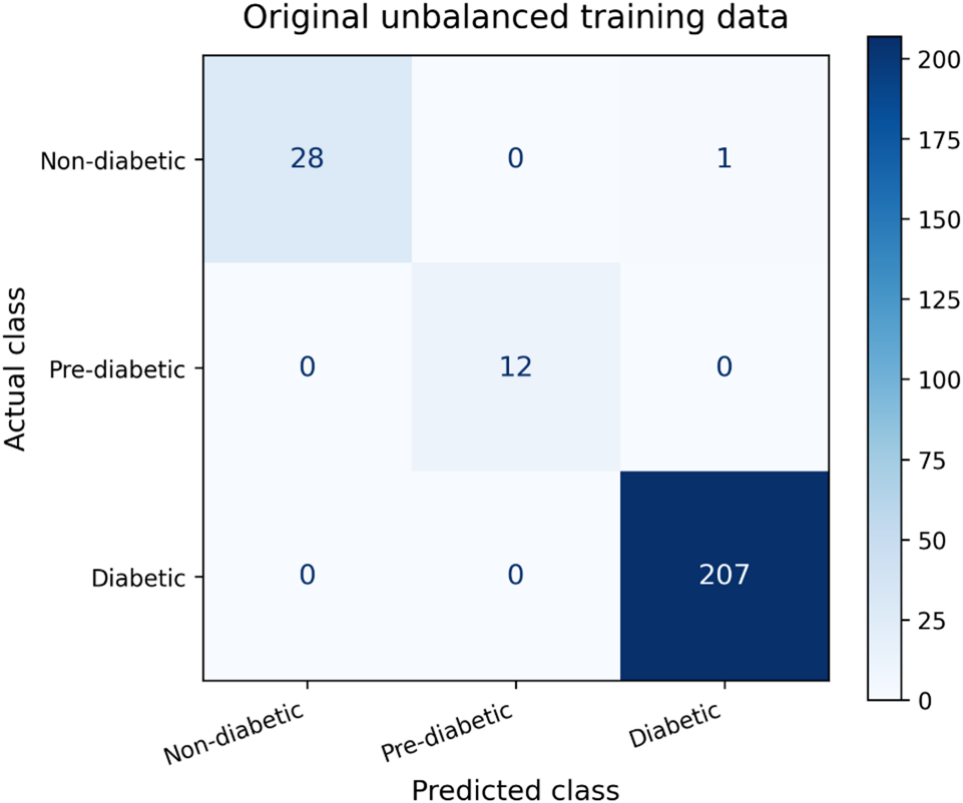
Confusion matrix of XGBoost trained on the original dataset.

**Fig. 5:**
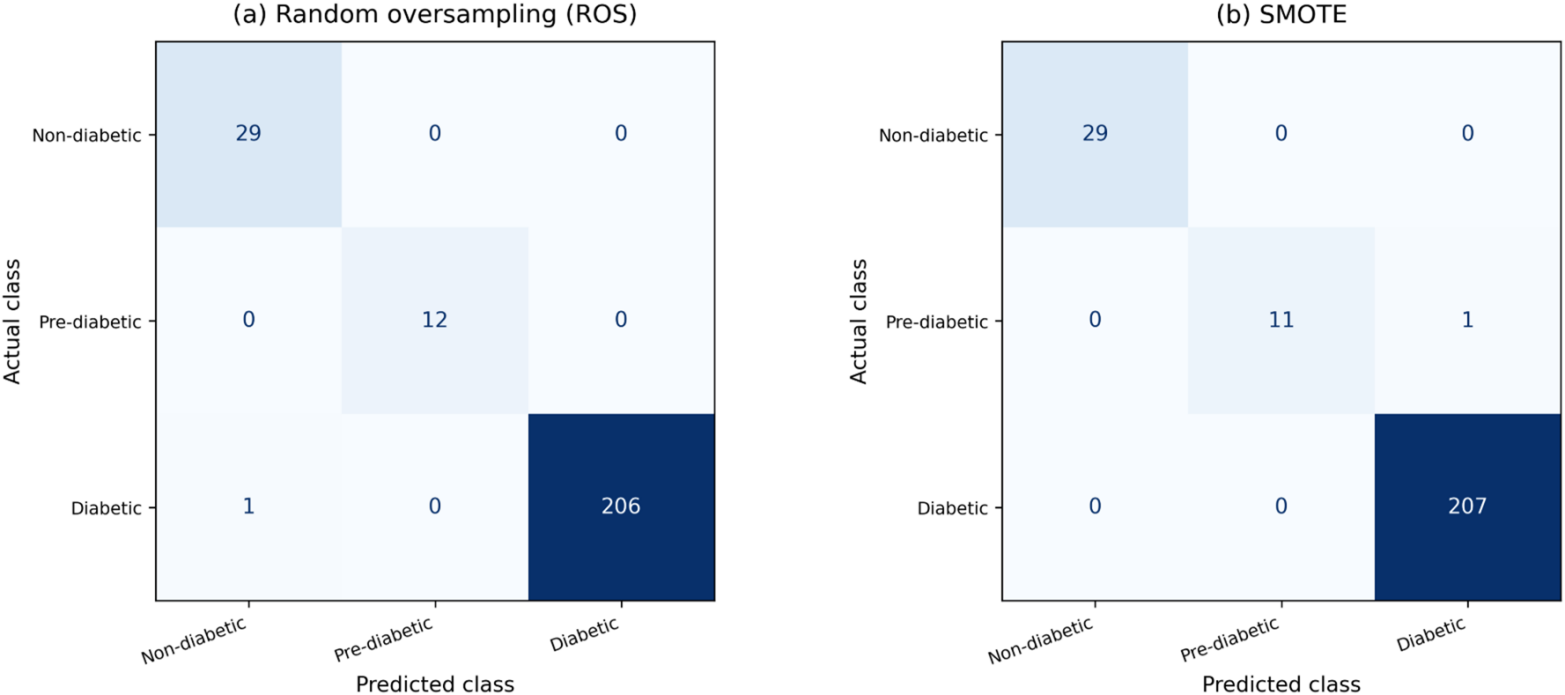
Confusion matrices of XGBoost after oversampling using (**a**) ROS and (**b**) SMOTE.

**Fig. 6:**
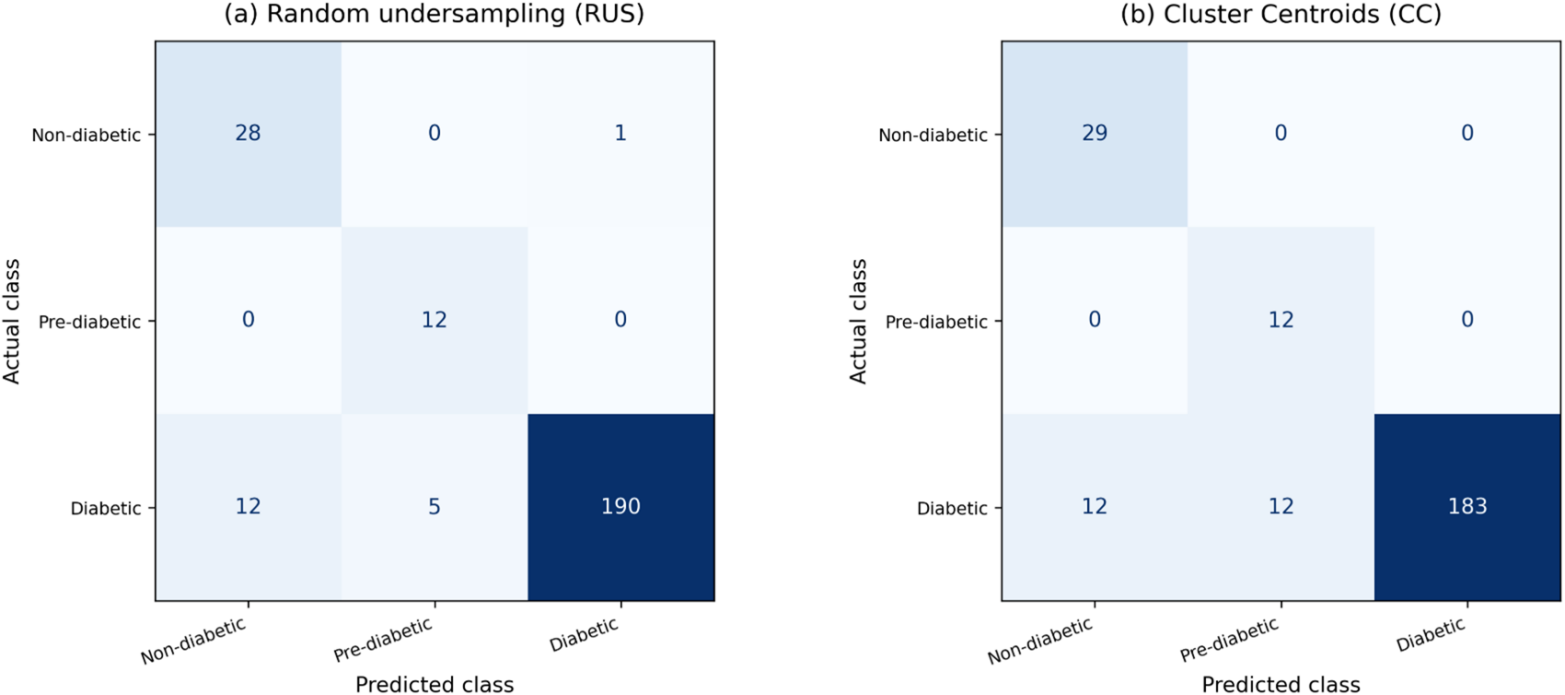
Confusion matrices of XGBoost after undersampling using (**a**) RUS and (**b**) Cluster Centroids.

Tables 6 and 7 compare XGBoost across the train-test split and five-fold cross validation. The proposed XGBoost model was compared with Logistic Regression, Random Forest, Support Vector Machine, Decision Tree, and K-Nearest Neighbors under the same experimental protocol. In the stratified 70/30 holdout evaluation, XGBoost achieved 99.60% accuracy, 99.84% precision, 98.85% recall, and 99.33% F1-score. Decision Tree reached the same holdout accuracy and slightly higher recall and F1-score; however, the five-fold cross-validation results provided stronger evidence of model stability and generalization. XGBoost obtained the highest mean cross-validation accuracy (98.79 ± 1.13%), precision (98.33 ± 1.65%), recall (97.22 ± 3.33%), and F1-score (97.72 ± 2.42%) among all benchmark classifiers. The closest competitor, Random Forest, achieved 98.30 ± 0.79% accuracy and 95.22 ± 2.14% F1-score, whereas Decision Tree achieved 97.46 ± 0.79% accuracy and 94.26 ± 2.55% F1-score. Logistic Regression, Support Vector Machine, and K-Nearest Neighbors showed substantially lower F1-scores. These findings indicate that XGBoost maintained a strong balance between overall accuracy and class-wise performance across independent folds, supporting the robustness of the proposed model for distinguishing non-diabetic, pre-diabetic, and diabetic cases. Tables 8 and 9 compare benchmark classifiers across the train-test split and five-fold cross validation under different resampling techniques. In the train-test evaluation, the unsampled, ROS, and SMOTE conditions each achieved 99.60% accuracy. RUS and Cluster Centroids reduced accuracy to 92.74% and 90.32%, respectively. Across five folds, the original XGBoost model achieved the highest mean accuracy of 98.79 ± 1.13%. Figures 4, 5 and 6 present the corresponding XGBoost train-test confusion matrices. Figure 4 shows the original dataset, Fig. 5 presents (a) ROS and (b) SMOTE, and Fig. 6 presents (a) RUS and (b) Cluster Centroids.

**Table 6:** Train-test performance of XGBoost under different resampling techniques.

Sampling Approach	Accuracy	Precision	Recall	F1-score	Run time (msec)
Original	0.9960	0.9984	0.9885	0.9933	85.57
ROS	0.9960	0.9889	0.9984	0.9935	102.45
SMOTE	0.9960	0.9984	0.9722	0.9847	117.24
RUS	0.9274	0.8002	0.9611	0.8647	49.36
CC	0.9032	0.7358	0.9614	0.8112	814.35

**Table 7:** Five-fold cross-validation performance of XGBoost (mean ± standard deviation) under different resampling techniques.

Sampling Approach	Accuracy	Precision	Recall	F1-score	Run time (msec)
Original	0.9879 ± 0.0113	0.9833 ± 0.0165	0.9722 ± 0.0333	0.9772 ± 0.0242	80.2049 ± 8.1119
ROS	0.9867 ± 0.0100	0.9666 ± 0.0192	0.9778 ± 0.0337	0.9712 ± 0.0206	105.7962 ± 3.6689
SMOTE	0.9867 ± 0.0079	0.9753 ± 0.0150	0.9778 ± 0.0248	0.9760 ± 0.0179	122.4580 ± 17.5151
RUS	0.9153 ± 0.0251	0.7798 ± 0.0578	0.9662 ± 0.0101	0.8419 ± 0.0489	53.8326 ± 5.9744
CC	0.8947 ± 0.0560	0.7419 ± 0.0717	0.9580 ± 0.0223	0.8086 ± 0.0732	739.5843 ± 45.1971

**Table 8:** Train-test comparison of all classifiers and different resampling techniques.

Classifier	Sampling approach	Accuracy	Precision	Recall	F1-score	Run time (msec)
LR	Original	0.9194	0.6573	0.6503	0.6381	9.68
	ROS	0.8992	0.7332	0.9301	0.7848	77.55
	SMOTE	0.9032	0.7313	0.8956	0.7776	78.45
	RUS	0.8871	0.7194	0.9055	0.7619	6.77
	CC	0.8669	0.6951	0.9073	0.7429	874.17
RF	Original	0.9718	0.9698	0.8742	0.9108	191.41
	ROS	0.9798	0.9730	0.9297	0.9487	145.97
	SMOTE	0.9879	0.9688	0.9952	0.9812	198.72
	RUS	0.9153	0.7523	0.9563	0.8215	92.24
	CC	0.9032	0.7350	0.9614	0.8077	746.85
SVM	Original	0.9032	0.5469	0.5782	0.5621	7.47
	ROS	0.9194	0.7747	0.8887	0.8118	17.73
	SMOTE	0.9194	0.7747	0.8887	0.8118	18.43
	RUS	0.8589	0.6892	0.8186	0.6983	4.50
	CC	0.8508	0.6817	0.8648	0.7152	675.79
DT	Original	0.9960	0.9889	0.9984	0.9935	4.17
	ROS	0.9919	0.9968	0.9607	0.9781	5.05
	SMOTE	0.9839	0.9575	0.9575	0.9575	9.40
	RUS	0.9032	0.7659	0.8922	0.8168	3.32
	CC	0.9395	0.8002	0.9758	0.8612	650.33
KNN	Original	0.8790	0.6740	0.6697	0.6697	5.90
	ROS	0.8669	0.6511	0.7695	0.6850	8.17
	SMOTE	0.8790	0.6906	0.8465	0.7330	10.32
	RUS	0.7379	0.5574	0.7343	0.5673	3.73
	CC	0.7742	0.5752	0.7685	0.6018	653.00
XGB	Original	0.9960	0.9984	0.9885	0.9933	85.57
	ROS	0.9960	0.9889	0.9984	0.9935	102.45
	SMOTE	0.9960	0.9984	0.9722	0.9847	117.24
	RUS	0.9274	0.8002	0.9611	0.8647	49.36
	CC	0.9032	0.7358	0.9614	0.8112	814.35

**Table 9:** Five-fold comparison of all classifiers and different resampling techniques (mean ± standard deviation).

Classifier	Sampling approach	Accuracy	Precision	Recall	F1-score	Run time (msec)
LR	Original	0.9213 ± 0.0096	0.7345 ± 0.1116	0.6764 ± 0.0355	0.6794 ± 0.0607	23.0914 ± 26.9196
	ROS	0.8995 ± 0.0176	0.7217 ± 0.0358	0.8643 ± 0.0628	0.7690 ± 0.0505	43.2022 ± 35.7240
	SMOTE	0.9055 ± 0.0128	0.7252 ± 0.0365	0.8619 ± 0.0648	0.7707 ± 0.0448	57.6527 ± 35.8574
	RUS	0.8704 ± 0.0205	0.6764 ± 0.0357	0.8299 ± 0.0625	0.7145 ± 0.0356	22.6388 ± 28.0646
	CC	0.8620 ± 0.0152	0.6650 ± 0.0173	0.8298 ± 0.0443	0.7044 ± 0.0156	739.6817 ± 53.2110
RF	Original	0.9830 ± 0.0079	0.9722 ± 0.0272	0.9401 ± 0.0435	0.9522 ± 0.0214	115.8953 ± 4.6716
	ROS	0.9855 ± 0.0069	0.9728 ± 0.0297	0.9519 ± 0.0294	0.9602 ± 0.0200	150.3652 ± 1.7677
	SMOTE	0.9782 ± 0.0092	0.9551 ± 0.0310	0.9539 ± 0.0241	0.9526 ± 0.0213	215.1295 ± 7.8501
	RUS	0.9237 ± 0.0126	0.7891 ± 0.0321	0.9558 ± 0.0199	0.8478 ± 0.0348	90.4436 ± 4.5922
	CC	0.8874 ± 0.0324	0.7229 ± 0.0535	0.9413 ± 0.0189	0.7859 ± 0.0486	781.1719 ± 45.4746
SVM	Original	0.9250 ± 0.0109	0.9067 ± 0.0347	0.6835 ± 0.0213	0.7153 ± 0.0398	10.8810 ± 0.4828
	ROS	0.9177 ± 0.0195	0.7723 ± 0.0698	0.8406 ± 0.0976	0.7968 ± 0.0763	25.7834 ± 0.6405
	SMOTE	0.9261 ± 0.0179	0.7979 ± 0.0689	0.8343 ± 0.0908	0.8100 ± 0.0779	27.2044 ± 2.0052
	RUS	0.8523 ± 0.0151	0.6473 ± 0.0327	0.7662 ± 0.0741	0.6626 ± 0.0242	8.5720 ± 0.6095
	CC	0.8378 ± 0.0093	0.6381 ± 0.0350	0.7872 ± 0.0744	0.6614 ± 0.0354	699.3879 ± 72.3410
DT	Original	0.9746 ± 0.0079	0.9532 ± 0.0317	0.9344 ± 0.0302	0.9426 ± 0.0255	7.9046 ± 0.7703
	ROS	0.9734 ± 0.0140	0.9462 ± 0.0200	0.9307 ± 0.0456	0.9355 ± 0.0230	10.9964 ± 3.8913
	SMOTE	0.9843 ± 0.0081	0.9637 ± 0.0313	0.9691 ± 0.0222	0.9649 ± 0.0169	13.2285 ± 0.5489
	RUS	0.9250 ± 0.0201	0.7962 ± 0.0501	0.9592 ± 0.0224	0.8534 ± 0.0351	7.1561 ± 0.1901
	CC	0.9068 ± 0.0314	0.7520 ± 0.0564	0.9491 ± 0.0242	0.8195 ± 0.0488	758.7807 ± 52.8242
KNN	Original	0.9116 ± 0.0134	0.7612 ± 0.1015	0.7005 ± 0.0973	0.7019 ± 0.0905	8.3139 ± 0.2360
	ROS	0.8983 ± 0.0204	0.7104 ± 0.0605	0.8001 ± 0.0530	0.7398 ± 0.0592	11.3094 ± 0.4762
	SMOTE	0.8850 ± 0.0115	0.6917 ± 0.0222	0.8221 ± 0.0460	0.7293 ± 0.0320	11.8737 ± 0.4295
	RUS	0.7616 ± 0.0510	0.5689 ± 0.0223	0.7118 ± 0.0678	0.5770 ± 0.0212	7.3217 ± 0.0673
	CC	0.7821 ± 0.0057	0.5715 ± 0.0230	0.7220 ± 0.0590	0.5879 ± 0.0208	679.4552 ± 80.0072
XGB	Original	0.9879 ± 0.0113	0.9833 ± 0.0165	0.9722 ± 0.0333	0.9772 ± 0.0242	80.2049 ± 8.1119
	ROS	0.9867 ± 0.0100	0.9666 ± 0.0192	0.9778 ± 0.0337	0.9712 ± 0.0206	105.7962 ± 3.6689
	SMOTE	0.9867 ± 0.0079	0.9753 ± 0.0150	0.9778 ± 0.0248	0.9760 ± 0.0179	122.4580 ± 17.5151
	RUS	0.9153 ± 0.0251	0.7798 ± 0.0578	0.9662 ± 0.0101	0.8419 ± 0.0489	53.8326 ± 5.9744
	CC	0.8947 ± 0.0560	0.7419 ± 0.0717	0.9580 ± 0.0223	0.8086 ± 0.0732	739.5843 ± 45.1971

### Ethics approval and consent to participate

All methods were carried out in accordance with relevant guidelines and regulations. This study utilized publicly available, fully anonymized data from the Mendeley Data repository (https://data.mendeley.com/datasets/wj9rwkp9c2/1), and did not involve direct interaction with human participants. Therefore, ethical approval and informed consent were not required.

## Discussion

The proposed model ensures that XGBoost provides strong multiclass discrimination after duplicate clinical records are removed. Where XGB yielded an accuracy 99.60% on original unbalanced dataset. Both SMOTE and ROS produced the same accuracy. Yet, the duplication of minority samples can increase risk of overfitting. The cross-validation results provide a more conservative assessment than the single test split. The XGB model achieved the highest mean accuracy of 98.79 ± 1.13% on original size dataset. SMOTE and ROS remained close, with mean accuracies of 98.67%, but neither consistently exceeded the original model. Thus, oversampling did not provide a clear overall advantage for XGB after duplicate removal and leakage control. By contrast, RUS and Cluster Centroids substantially reduced accuracy. Both methods increased minority-class sensitivity but generated additional false diabetic predictions because the majority class was compressed. Cluster Centroids also required the longest runtime because centroid construction introduced a clustering stage before model fitting. The benchmark experiments clarify that a very high score on one holdout split is not sufficient to establish model superiority. Decision Tree and XGBoost both achieved 99.60% holdout accuracy on the original data, but XGBoost produced the strongest five-fold accuracy, indicating greater stability across data partitions. Random Forest was the next strongest benchmark. Logistic Regression and Support Vector Machine benefited markedly from ROS or SMOTE in F1-score, showing that balancing can improve minority-class recognition for models whose unbalanced decision boundaries favor the diabetic majority. Decision Tree also benefited from SMOTE during cross-validation, whereas KNN remained less effective across most conditions. Clinically, three-class prediction may support screening by distinguishing individuals without diabetes from those who may require preventive assessment and those with laboratory patterns consistent with diabetes. Correct identification of the pre-diabetic group is particularly important because it can motivate early lifestyle intervention and confirmatory testing. False-negative predictions may delay assessment, whereas false-positive predictions may cause unnecessary anxiety or testing. The proposed system should therefore be considered a decision-support or screening model rather than an independent diagnostic instrument. The study has several limitations. The final sample contains only 40 pre-diabetic records, the diabetic class represents 83.54% of the dataset, and the data originate from specific Iraqi clinical centers. Detailed patient-level inclusion criteria and independent external cohorts are unavailable. Although duplicate removal and leakage-controlled cross-validation improve internal validity, they do not establish generalizability to other institutions, populations, laboratory protocols, or prospective clinical settings. External validation on larger and geographically diverse datasets is required before clinical deployment.

## Conclusion

This study developed a reproducible XGBoost framework for multi-classification of non-diabetic, pre-diabetic, and diabetic records. After standardizing categorical labels, excluding identifier fields, and removing 174 duplicate clinical records, 826 unique observations were evaluated using a stratified holdout design and stratified five-fold cross-validation. The XGBoost model achieved 99.60% holdout accuracy and 98.79 ± 1.13% cross-validated accuracy on the original unbalanced dataset. ROS and SMOTE maintained high performance, but neither consistently improved XGBoost across folds. RUS and Cluster Centroids reduced predictive performance because majority-class information was removed or compressed. Across conventional benchmark classifiers, XGBoost provided the strongest cross-validated accuracy and F1-score, while the full classifier-sampling comparison showed that oversampling was more beneficial for Logistic Regression, Support Vector Machine, and selected tree models than for XGBoost. Future research may focus on validating the proposed model using larger clinical datasets to extend the generalization capability of the proposed model. Additionally, hybrid imbalance-handling could be investigated to further improve minority subtype detection, particularly in multiclass scenarios where certain diabetes subtypes are underrepresented. Lastly, integrating explainable artificial intelligence (XAI) techniques may enhance interpretability by quantifying individual feature contributions to model predictions. This would increase transparency and facilitate clinical adoption by providing physicians with actionable insights into risk factors influencing each classification outcome.

## Data Availability

The dataset used in this study is publicly available from the Mendeley Data repository (https://data.mendeley.com/datasets/wj9rwkp9c2/1). All data are accessible without restriction. Additional details can be obtained from the corresponding author upon reasonable request.
